# Methodological quality of systematic reviews on treatments for Alzheimer’s disease: a cross-sectional study

**DOI:** 10.1186/s13195-022-01100-w

**Published:** 2022-10-29

**Authors:** Claire C. W. Zhong, Jinglun Zhao, Charlene H. L. Wong, Irene X. Y. Wu, Chen Mao, Jerry W. F. Yeung, Vincent C. H. Chung

**Affiliations:** 1 Jockey Club School of Public Health and Primary Care, The Chinese University of Hong Kong, Shatin, Hong Kong; 2 Xiangya School of Public Health, Central South University, 5/F, No. 238, Shang ma Yuan ling Alley, Kaifu District, Changsha, Hunan China; 3 Department of Epidemiology, School of Public Health, Southern Medical University, Guangzhou, China; 4 School of Nursing, The Hong Kong Polytechnic University, Hung Hom, Hong Kong; 5 School of Chinese Medicine, The Chinese University of Hong Kong, Shatin, Hong Kong

**Keywords:** Alzheimer’s disease, Cross-sectional study, Meta-analysis, Bias, Systematic reviews

## Abstract

**Background:**

Carefully conducted systematic reviews (SRs) can provide reliable evidence on the effectiveness of treatment strategies for Alzheimer’s disease (AD). Nevertheless, the reliability of SR results can be limited by methodological flaws. This cross-sectional study aimed to examine the methodological quality of SRs on AD treatments, along with potentially relevant factors.

**Methods:**

To identify eligible SRs on AD treatments, four databases including the Cochrane Database of Systematic Reviews, MEDLINE, EMBASE, and PsycINFO were searched. The Assessing the Methodological Quality of Systematic Reviews 2 instrument was used for quality appraisal of SRs. Multivariable regression analyses were used to examine factors related to methodological quality.

**Results:**

A total of 102 SRs were appraised. Four (3.90%) SRs were considered as high quality; 14 (13.7%), 48 (47.1%), and 36 (35.3%) were as moderate, low, and critically low quality, respectively. The following significant methodological limitations were identified: only 22.5% of SRs registered protocols a priori, 6.9% discussed the rationales of chosen study designs, 21.6% gave a list of excluded studies with reasons, and 23.5% documented funding sources of primary studies. Cochrane SRs (adjusted odds ratio (AOR): 31.9, 95% confidence interval (CI): 3.81–266.9) and SRs of pharmacological treatments (AOR: 3.96, 95%CI: 1.27–12.3) were related to the higher overall methodological quality of SRs.

**Conclusion:**

Methodological quality of SRs on AD treatments is unsatisfactory, especially among non-Cochrane SRs and SRs of non-pharmacological interventions. Improvement in the following methodological domains requires particular attention due to poor performance: registering and publishing protocols a priori, justifying study design selection, providing a list of excluded studies, and reporting funding sources of primary studies.

**Supplementary Information:**

The online version contains supplementary material available at 10.1186/s13195-022-01100-w.

## Introduction

Alzheimer’s disease (AD) is the most common type of dementia and one of the major causes of mortality globally [1], with a life expectancy of 7–10 years among AD patients aged 60s to early 70s [2]. It is characterized by progressive deterioration of cognitive function and memory loss, disturbances in activities of daily living, various neuropsychiatric symptoms, and behavioral disorders [3]. In the USA, approximately 6.2 million people aged 65 years or above live with AD, with a total healthcare expenditure estimated at $355 billion in 2021 [3]. Given the increasing proportion of population aged 65 years or above, the prevalence of AD is expected to double by 2050, and it is projected to reach 13.8 million in 2060 [3, 4]. The increasing trend of AD prevalence and long duration of illness before death significantly increase the burden of AD, leading to a tremendous challenge to health and social care systems worldwide.

Despite massive efforts to develop a successful treatment, there is still no cure for AD today. Managing neuropsychiatric symptoms, optimizing physical health, cognition, activity, well-being, preventing behavioral or psychological disorders, and providing information and long-term support to caregivers are key goals for AD management [5]. Currently, both pharmacological treatments and non-pharmacological interventions are essential in the management of AD [3]. With the availability of different treatment options, it is crucial for healthcare professionals to access the best available clinical evidence, and select the most appropriate treatments for AD patients.

Results from carefully-conducted systematic reviews (SRs) are considered to provide the highest level of evidence on the effectiveness of different AD treatments. According to the Cochrane handbook version 6, an SR identifies, critically appraises, and synthesizes evidence from eligible studies that fulfill predefined eligibility criteria using explicit and systematic methods [6]. If appropriate, a meta-analysis could be conducted to synthesize quantitative effect estimates and investigate heterogeneity among included studies [6]. In recent years, the number of SRs has been increasing exponentially. However, not all SRs are methodologically rigorous [7]. SRs with methodological flaws may exaggerate or underestimate treatment effects, consequently misleading clinical decision-making. Therefore, evidence users, including policymakers, healthcare professionals, caregivers, and patients, should evaluate the methodological quality of SRs carefully before implementing the findings and conclusions into practice.

Currently, a comprehensive evaluation of the methodological quality of SRs in AD treatments has not been conducted. Therefore, in this cross-sectional analysis, we sought to (i) evaluate the methodological quality of SRs using the validated Assessing the Methodological Quality of Systematic Reviews 2 (AMSTAR2) instrument [8], (ii) outline the bibliographic features of an up-to-date sample of SRs on AD treatments, and (iii) investigate the associations between methodological quality and bibliographic characteristics. Findings of this study could provide insights on how to improve the methodological quality of future SRs on AD treatments.

## Methods

### Eligibility criteria

In this cross-sectional study, eligible SRs should focus on randomized controlled trials (RCTs), and include one or more meta-analyses that synthesize the effectiveness of AD treatments. SRs synthesizing results of RCTs recruiting AD patients were considered eligible, while SRs only focusing on other clinical forms of dementia or mild cognitive impairment were excluded. Any pharmacological treatments and non-pharmacological interventions for AD were accepted. SRs focusing on diagnostics, etiology, or risk factors, network meta-analyses, narrative reviews, overviews of SRs, and protocols were excluded. Language was restricted to English. SRs published before 2014 were excluded, as the median time for updating the conclusion of an SR was 5.5 years [9]. Appraising out-of-date SRs is not a goal of this study as they do not provide accurate information for decision-making. The latest version of an SR was included when multiple versions were available.

### Literature search, literature selection, data extraction, and methodological quality assessment

We searched for potential SRs in four electronic databases: MEDLINE, EMBASE, PsycINFO, and Cochrane Database of Systematic Reviews from January 2014 to February 2021. When searching for SRs in MEDLINE [10], EMBASE [11], and PsycINFO [12], specialized search filters were used to enhance search specificity. Detailed search strategies are listed in Additional file 1: Appendix 1.

Two reviewers (CZ and JZ) screened the title and abstract of retrieved citations and further assessed the full text for potentially eligible SRs independently. Then, they independently extracted details of bibliographic characteristics of included SRs using a pre-designed data extraction form (Additional file 1: Appendix 2). Discrepancies were settled by discussion. A third reviewer (CW) was consulted when disagreements persisted.

For methodological quality appraisal, the AMSTAR2 instrument, which comprised 16 items, was adopted. Among these 16 items, seven were critical items (items 2, 4, 7, 9, 11, 13, 15) [8]. Based on information reported in the full text of SRs, items 1, 3, 5–6, and 10–16 were appraised as “yes” or “no,” while items 2, 4, and 7–9 were appraised as “yes,” “partial yes,” or “no” [8]. Overall methodological quality was an overall rating of methodological quality for each included SR, which was generated based on the assessment results of the 16 AMSTAR2 items. The overall methodological quality could be rated as “critically low,” “low,” “moderate,” or “high” [8]. Detailed information on AMSTAR2 items and the operational guidance for defining each rank of quality can be found in Additional file 1: Appendix 3. The quality appraisal was also conducted by two reviewers independently (JZ and CZ), with disagreements resolved by discussion and consensus, or by consulting a third reviewer (CW) if disagreements persisted.

### Data analysis

Descriptive variables including bibliographic characteristics and methodological quality were reported as frequencies with percentages and medians with ranges, as appropriate. The Kruskal-Wallis rank test was applied to evaluate the differences in overall methodological quality across SRs with varied bibliographic features.

Based on findings from previous methodological research [13–15], seven bibliographic characteristics of SRs were selected as independent variables in regression analyses to investigate their potential association to the rigor of SRs, including (i) whether the SR was a Cochrane SR; (ii) whether the SR was an update of a previous SR; (iii) types of treatment; (iv) publication year; (v) publication journal impact factor in the year before SR publication; (vi) number of authors in the SR; and (vii) location of the corresponding author. To examine potential associations between overall methodological quality and bibliographic characteristics, multi-ordinal regressions were conducted. Associations between ratings of individual AMSTAR2 items and bibliographic characteristics were further examined using either binary logistic regression (for items 1, 3, 5, 6, and 10–16) or multinomial logistic regression (items 2, 4, and 7–9). In multi-ordinal regression analysis, binary logistic regression analysis, and multinomial logistic regression analysis, the model fit was evaluated using the Pearson/deviance test, Hosmer-Lemeshow test, and likelihood ratio test. Adjusted odds ratios (AORs) and 95% confidence intervals (CIs) were calculated to quantify the associations between AMSTAR2 ratings and bibliographic characteristics. A *p* <0.05 indicated statistical significance. Data analyses were conducted by IBM SPSS v26.

## Results

### Literature screening and selection

**Fig. 1:**
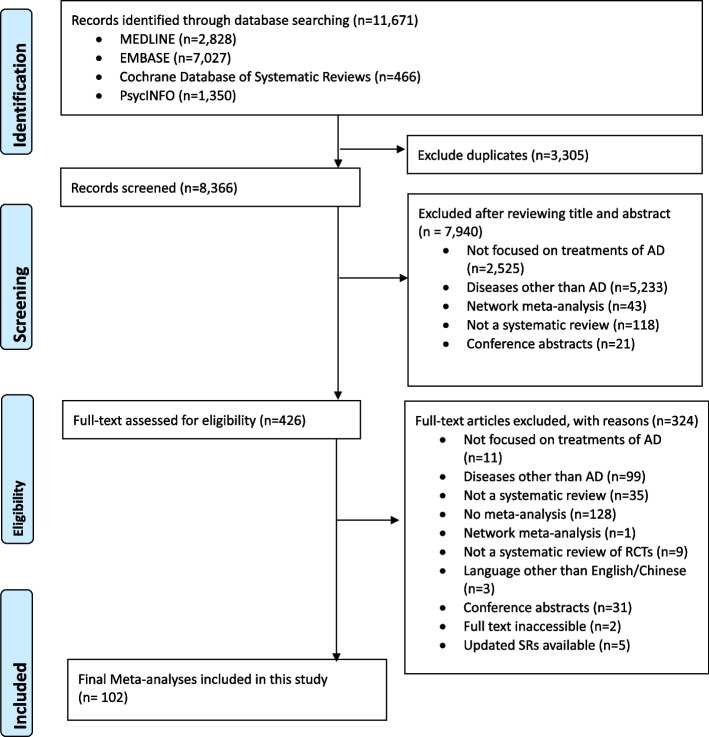
Flowchart of literature search and selection for systematic reviews on treatments of Alzheimer’s disease. Notes: RCTs, randomized controlled trials; AD, Alzheimer’s disease; SR: Systematic review

Through literature search, we identified a total of 11,671 records. After deduplication, 8366 records had their title and abstract screened, 426 of which were screened full text for eligibility. Finally, a total of 102 SRs were considered eligible and included. The list of included SRs is presented in Additional file 1: Appendix 4. Detailed information on literature search and screening is shown in Fig. 1.

### Bibliographic characteristics

Table 1 depicts the characteristics of the 102 SRs. They included a total of 1158 RCTs, which recruited 244,216 AD patients. For the number of included RCTs in SRs, the median number is 7, with a range of 2–69. The median journal impact factor in the year before SR publication was 3.26 (range, 0.17–8.77). Most of them (92 SRs, 90.2%) were non-Cochrane SRs. Only 14 (13.7%) were an update of a previous SR. Corresponding authors of the included SRs were mostly from Asia (68 SRs, 66.7%), Europe (18 SRs, 17.6%), and America (12 SRs, 11.8%). About 21.6% of the included SRs did not report whether they received funding or not, while 20.6% reported receiving no funding support. For those with funding support reported, the funding location of most SRs was in Asia (35 SRs, 34.3%). Around 14.7% were in Europe. Treatments for AD covered across pharmacological treatments (77 SRs, 75.5%), non-pharmacological interventions (19 SRs, 18.6%), and both types (6 SRs, 5.9%). Most SRs of pharmacological treatments (65 SRs, 84.4%) reported harm. However, only 11 SRs (57.9%) of non-pharmacological interventions did so.

**Table 1:** Bibliographical characteristics of 102 included systematic reviews

Bibliographical characteristics	Results^**a**^
**Cochrane SRs**	
- Yes	10 (9.8)
- No	92 (90.2)
**An update of a previous SR**	
- Yes	14 (13.7)
- No	88 (86.3)
**Publication year**	
2014–2015	36 (35.3)
2016–2018	29 (28.4)
2019–2021	37 (36.3)
**Publication journal impact factor in the year before SR publication, median [range]**	3.26 [0.17,8.77]
**Number of review authors, median [range]**	5 [2,17]
**Location of the corresponding author**	
- Europe	18 (17.6)
- America	12 (11.8)
- Asia	68 (66.7)
- Oceania	3 (2.9)
- Africa	1 (1.0)
**Funding location of the SR**	
- Europe	15 (14.7)
- America	7 (6.9)
- Asia	35 (34.3)
- Oceania	2 (2.0)
- No funding	21 (20.6)
- Not reported	22 (21.6)
**Type of treatment**	
- Pharmacological treatments	77 (75.5)
- Non-pharmacological interventions	19 (18.6)
- Both types	6 (5.9)
**Total number of included primary studies**	1158
**Median number of included primary studies[range]**	7 [2,69]
**Total number of participants included in primary studies**	244,216
**Median number of participants included in primary studies[range]**	926.5 [57,20132]
**SRs reporting intervention harms**^**b**^	80 (78.4)
Pharmacological treatments	65 (84.4)
Non-pharmacological interventions	11 (57.9)
Both types	4 (66.7)
**SRs that search for English databases**	102 (100)
**SRs that search for non-English databases**	
- Yes	51 (50.0)
- No	51 (50.0)
**Included a PRISMA-like flow diagram**	
- Yes	87 (85.3)
- No	15 (14.7)
**Search terms reported for electronic databases**	
- No search terms reported	2 (2.0)
- Topics/free text/keywords/MeSH	61 (59.8)
- Full Boolean	37 (36.3)
- Readers are referred elsewhere for full search strategy	2 (2.0)
**Report year span of search**	
- Yes, reported both starting and ending years	50 (49.0)
- Partially (only reported starting year or ending year)	49 (48.0)
- Not mentioned	3 (2.9)
**Language restrictions of primary studies**	
- English only	24 (23.5)
- Language other than English	1 (1.0)
- English and language other than English	53 (52.0)
- Not reported	24 (23.5)
**Methodological quality assessment tools**	
- Cochrane risk of bias tool	67 (65.7)
- Jadad scale	8 (7.8)
- Pedro Scale	1 (1.0)
- Delphi list	1 (1.0)
- More than one tools	6 (5.9)
- Other tools	7 (6.9)
- Name of the assessment tool not reported	7 (6.9)
- No quality assessment conducted	5 (4.9)

*SR* systematic review, *PRISMA* Preferred Reporting Items for Systematic Reviews and Meta-analysis, *MeSH* Medical Subject Headings

^a^Values are *N* (%) or median [range]

^b^Percentages were calculated by using the total number of the categories as the denominator

All of the included SRs searched English databases, whereas half SRs additionally searched non-English databases. Most SRs (87 SRs, 85.3%) included a PRISMA flowchart. Twenty-four (23.5%) of SRs did not state language restrictions for primary studies. Over half (67 SRs, 65.7%) used the Cochrane Risk of Bias Tool for assessing the quality of primary studies. Five SRs (4.9%) did not use any tools for appraising risk of bias.

### Overall methodological quality

For each included SR, detailed results on each AMSTAR2 item and the overall methodological quality are presented in Additional file 1: Appendix 5. For overall methodological quality, only 4 SRs (3.9%) were deemed to have high quality. The remaining 14 (13.7%), 48 (47.1%), and 36 (35.3%) were of moderate, low, and critically low quality, respectively. Kruskal-Wallis test results revealed significant differences in overall quality among different bibliographical characteristic groups (Table 2).

**Table 2:** Overall methodological quality of the included systematic reviews by bibliographical characteristics

AMSTAR2 rated overall methodological quality^**b**^	Critically low^**a**^	Low^**a**^	Moderate^**a**^	High^**a**^	***P*** values
Total	36 (35.3)	48 (47.1)	14 (13.7)	4 (3.9)	-
**Cochrane SRs**					<0.001***
Yes	2 (20.0)	0 (0)	4 (40.0)	4 (40.0)	
No	34 (37.0)	48 (52.2)	10 (10.9)	0 (0)	
**An update of a previous SR**					0.605
Yes	5 (35.7)	5 (35.7)	3 (21.4)	1 (7.1)	
No	31 (35.2)	43 (48.9)	11 (12.5)	3 (3.4)	
**SRs reporting intervention harms**					0.188
Yes	27 (33.8)	36 (45.0)	13 (16.3)	4 (5.0)	
No	9 (40.9)	12 (54.5)	1 (4.5)	0 (0)	
**SRs that search for non-English databases**					0.085
Yes	15 (29.4)	24 (47.1)	8 (15.7)	4 (7.8)	
No	21 (41.2)	24 (47.1)	6 (11.8)	0 (0)	
**Included a PRISMA-like flow diagram**					0.086
Yes	30 (34.5)	42 (48.3)	11 (12.6)	4 (4.6)	
No	6 (40.0)	6 (40.0)	3 (20.0)	0 (0)	
**Publication year**					0.652
2014–2015	15 (41.7)	15 (41.7)	6 (16.7)	0 (0)	
2016–2018	8 (27.6)	18 (62.1)	1 (3.4)	2 (6.9)	
2019–2021	13 (35.1)	15 (40.5)	7 (18.9)	2 (5.4)	
**Location of the corresponding author**					0.016*
Europe	3 (16.7)	7 (38.9)	5 (27.8)	3 (16.7)	
America	6 (50.0)	4 (33.3)	1 (8.3)	1 (8.3)	
Asia	27 (39.7)	34 (50.0)	7 (10.3)	0 (0)	
Oceania	0 (0)	3 (100)	0 (0)	0 (0)	
Africa	0 (0)	0 (0)	1 (100)	0 (0)	
**Funding reported by the SR**					0.240
No funding	6 (28.6)	11 (52.4)	4 (19.0)	0 (0)	
Yes	20 (33.9)	26 (44.1)	9 (15.3)	4 (6.8)	
Not reported	10 (45.5)	11 (50.0)	1 (4.5)	0 (0)	
**Types of treatment**					0.024*
Non-pharmacological interventions	11 (57.9)	8 (42.1)	0 (0)	0 (0)	
Pharmacological treatments	24 (31.2)	36 (46.8)	13 (16.9)	4 (5.2)	
Both types	1 (16.7)	4 (66.7)	1 (16.7)	0 (0)	
**Report year span of search**					0.854
Not mentioned	2 (66.7)	0 (0)	1 (33.3)	0 (0)	
Partially	19 (38.8)	19 (38.8)	7 (14.3)	4 (8.2)	
Yes	15 (30.0)	29 (58.0)	6 (12.0)	0 (0)	
**Search terms reported for electronic databases**					0.023*
No research term	0 (0)	2 (100)	0 (0)	0 (0)	
Topics/free text/keywords/MeSH	26 (42.6)	30 (49.2)	5 (8.2)	0 (0)	
Full Boolean	9 (24.3)	15 (40.5)	9 (24.3)	4 (10.8)	
Readers are referred elsewhere for full search strategy	1 (50)	1 (50)	0 (0)	0 (0)	
**Language criteria of primary studies**					0.240
English only	13 (54.2)	8 (33.3)	3 (12.5)	0 (0)	
Language other than English	0 (0)	1 (100)	0 (0)	0 (0)	
English and language other than English	14 (26.4)	32 (60.4)	4 (7.5)	3 (5.7)	
Not reported	9 (37.5)	7 (29.2)	7 (29.2)	1 (4.2)	
**Methodological quality assessment tools**					0.001**
Cochrane Risk of Bias	15 (22.4)	37 (55.2)	11 (16.4)	4 (6.0)	
Tools other than Cochrane Risk of Bias	11 (47.8)	10 (43.5)	2 (8.7)	0 (0)	
Names of the tools not reported	5 (71.4)	1 (14.3)	1 (14.3)	0 (0)	
No quality assessment	5 (100)	0 (0)	0 (0)	0 (0)	

*SR* systematic review, *PRISMA* Preferred Reporting Items for Systematic Reviews and Meta-analysis, *MeSH* Medical Subject Headings

*p-value of the Kruskal-Wallis test was <0.05, **p-value of the Kruskal-Wallis test was <0.01, ***p-value of the Kruskal-Wallis test was <0.001

^a^Values are *N* (% in subgroup)

^b^Overall methodological quality was an overall rating of methodological quality for each included SR, which was generated based on the assessment results of the 16 AMSTAR2 items. Detailed operational guidance for defining each rank of quality can be found in Additional file 1: Appendix 3

### Individual AMSTAR2 item performance

Performances of each AMSTAR2 item are presented in Table 3. The included SRs generally performed well on 6 out of 16 AMSTAR2 items, with over 80% being rated as satisfactory: (i) All SRs reported clear PICO information (Population, Intervention, Comparison, Outcome) in both research questions and inclusion criteria (item 1); (ii) 89 (87.3%) SRs used satisfactory techniques to assess risk of bias among primary studies (item 9); (iii) 97 (95.1%) SRs used appropriate methods for meta-analysis (item 11); (iv) 83 (81.4%) SRs provided satisfactory explanations for heterogeneity (item 14); (v) 92 (90.2%) SRs investigated publication bias and discussed potential impact (item 15); and (vi) all SRs reported potential conflicts of interest (item 16). Three of the criteria listed above (item 9, item 11, and item 15) are critical AMSTAR2 items.

**Table 3:** Performance on each individual AMSTAR-2 item

AMSTAR 2 items	Yes (%)	Partial yes (%)	No (%)
1. Did the research questions and inclusion criteria for the review include the components of PICO?	102 (100)	N/A	0 (0)
2. ^a^Did the report of the review contain an explicit statement that the review methods were established prior to the conduct of the review and did the report justify any significant deviations from the protocol?	23 (22.5)	73(71.6)	6 (5.9)
3. Did the review authors explain their selection of the study designs for inclusion in the review?	7 (6.9)	N/A	95 (93.1)
4. ^a^Did the review authors use a comprehensive literature search strategy?	43 (42.2)	58 (56.9)	1 (1.0)
5. Did the review authors perform study selection in duplicate?	58 (56.9)	N/A	44 (43.1)
6. Did the review authors perform data extraction in duplicate?	73 (71.6)	N/A	29 (28.4)
7. ^a^Did the review authors provide a list of excluded studies and justify the exclusions?	22 (21.6)	3 (2.9)	77 (75.5)
8. Did the review authors describe the included studies in adequate detail?	71 (69.6)	31 (30.4)	0 (0)
9. ^a^Did the review authors use a satisfactory technique for assessing the RoB in individual studies that were included in the review?	89 (87.3)	4 (3.9)	9 (8.8)
10. Did the review authors report on the sources of funding for the studies included in the review?	24 (23.5)	N/A	78 (76.5)
11. ^a^If meta-analysis was performed did the review authors use appropriate methods for statistical combination of results?	97 (95.1)	N/A	5 (4.9)
12. If meta-analysis was performed, did the review authors assess the potential impact of RoB in individual studies on the results of the meta-analysis or other evidence synthesis?	40 (39.2)	N/A	62 (60.8)
13. ^a^Did the review authors account for RoB in individual studies when interpreting/discussing the results of the review?	71 69.6)	N/A	31 (30.4)
14. Did the review authors provide a satisfactory explanation for, and discussion of, any heterogeneity observed in the results of the review?	83 (81.4)	N/A	19 (18.6)
15. ^a^If they performed quantitative synthesis did the review authors carry out an adequate investigation of publication bias (small study bias) and discuss its likely impact on the results of the review?	92 (90.2)	N/A	10 (9.8)
16. Did the review authors report any potential sources of conflict of interest, including any funding they received for conducting the review?	100 (98.0)	N/A	2 (2.0)

*AMSTAR* Assessing the Methodological Quality of Systematic Reviews, *N/A* not applicable, *PICO* patients, intervention, comparison, and outcomes, *RoB* risk of bias

^a ^Critical methodological items

Performance on the following items was considered unsatisfactory as less than 30% of SRs meet these quality criteria: (i) only 23 (22.5%) SRs registered protocols a priori with justification on deviations, if necessary (item 2), (ii) 7 (6.9%) SRs explained the selection of study designs for inclusion (item 3); (iii) 22 (21.6%) SRs provided a list of excluded studies with justifications (item 7); and (iv) 24 (23.5%) SRs reported funding sources of primary studies (item 10). Among these, item 2 and item 7 are critical AMSTAR2 items.

### Factors associated with methodological quality

According to multi-ordinal logistic regression results, the following characteristics of SRs contributed to a higher overall quality (Table 4). First, being a Cochrane SR could have a higher overall quality than a non-Cochrane SR, with an AOR of 31.9 and a 95% confidence interval (CI) of 3.81–266.9. Second, SRs of pharmacological treatments were of higher overall quality than non-pharmacological interventions (AOR 3.96, 95% CI 1.27–12.3). Both the Pearson test (*p* = 0.303) and the deviance test (*p* = 1.000) showed good model fitting.

**Table 4:** Associations between bibliographical characteristics of systematic reviews and overall methodological quality: multi-ordinal regression^f^

Bibliographical characteristics (independent variable)	AOR (95% CI)	***P-values***^**¶**^
**Cochrane SRs**^**a**^	**31.9 (3.81–266.9)**	**0.001**
An update of a previous SR^b^	0.35 (0.10–1.27)	0.111
Year of publication^c^		
2019–2021	2.43 (0.91–6.49)	0.076
2016–2018	1.87 (0.67–5.17)	0.230
Number of review authors	1.06 (0.91–1.22)	0.464
Publication journal impact factor in the year before SR publication	1.11 (0.84–1.47)	0.458
Types of treatment^d^		
Both types	3.37 (0.46–24.6)	0.230
**Pharmacological treatments**	**3.96 (1.27–12.3)**	**0.017**
Location of corresponding author^e^		
Corresponding author from Africa	10.6 (0.16–704.0)	0.272
Corresponding author from Oceania	0.58 (0.04–7.88)	0.681
Corresponding author from Asia	0.40 (0.11–1.47)	0.167
Corresponding author from America	0.29 (0.06–1.45)	0.131

*AOR* adjusted odds ratio, *CI* confidence interval, *SR* systematic review

¶ *P* values for both Pearson and deviance tests > 0.1 (Pearson test: *p*=0.303, deviance test: *p*=1.000), indicating good-model-fit

^a^Non-Cochrane SRs were used as reference

^b^SRs that are not updates of previous reviews were used as reference

^c^Year of publication was divided into three periods (2014–2015, 2016–2018, 2019–2021). SRs published in 2014–2015 were used as reference

^d^SRs of non-pharmacological interventions were used as reference

^e^SRs led by the corresponding author from Europe were used as reference

^f^Overall methodological quality was an overall rating of methodological quality for each included SR, which was generated based on the assessment results of the 16 AMSTAR2 items. Detailed operational guidance for defining each rank of quality can be found in Additional file 1: Appendix 3

Regression results assessing the associations between specific bibliographical factors and individual AMSTAR-2 item performance are shown in Additional file 1: Appendix 6a–6b. Compared to non-Cochrane SRs, Cochrane SRs performed significantly better in explaining study design selection (AOR, 13.0; 95% CI, 1.84–91.80), and reporting funding sources of primary studies (AOR, 30.6; 95% CI, 3.58–261.4). SRs of pharmacological treatments (AOR, 7.44; 95% CI, 1.49–37.3) and both types of treatments (AOR, 16.5; 95% CI, 1.44–188.5) showed better performance in assessing the potential impact of bias originating from primary studies than those of non-pharmacological interventions. Compared to SRs led by European corresponding authors, those led by Asian corresponding authors were less likely to account for primary study risk of bias when interpreting results (AOR, 0.19; 95% CI, 0.06–0.62). SRs published in journals with higher impact factors were more likely to provide satisfactory explanations for heterogeneity (AOR, 1.50; 95% CI, 1.04–2.18). All Hosmer-Lemeshow tests had *p* values larger than 0.1, indicating that all binary logistic regressions were well-fitted.

SRs with the following features were more likely to receive “Yes” rather than “Partial yes” ratings in the following aspects: (i) mentioning protocols a priori and justifying deviations (item 2) if the SRs were published between 2019 and 2021, led by European corresponding authors, and focused on both types of treatments; (ii) conducting a comprehensive literature search (item 4) if SRs were led by America corresponding authors or SRs of pharmacological treatments. SRs were more likely to be rated as “Yes” rather than “No” in providing a list of excluded studies with justifications (item 7) if the SRs were led by European corresponding authors or SRs of pharmacological treatments. We observed that *p* values were >0.1 for all Pearson and deviance tests, and *p* values were <0.05 for all likelihood ratio tests. All multinomial regression analyses showed good model-fitting.

## Discussion

### Summary of findings

This study appraises the methodological quality of an up-to-date sample of 102 SRs on AD treatments published between 2014 and 2021, covering both pharmacological treatments and non-pharmacological interventions, as well as Cochrane and non-Cochrane SRs. Our results indicate that the methodological quality of included SRs is unsatisfactory. Only 4 (3.9%) SRs are of high overall quality; 14 (13.7%) of moderate overall quality; 48(47.1%) of low overall quality; and the remaining 36 (35.3%) of critically low overall quality. For the four SRs with high overall methodological quality, their unique feature is that they have no flaws in the AMSTAR2 critical items listed in Additional file 1: Appendix 3. However, for most appraised SRs, there is still much room for improvement, particularly in the registration of protocols a priori, the explanation of study design selection, the provision of a list for excluded studies with justifications, and the reporting of funding sources among primary studies.

Cochrane SRs and SRs of pharmacological treatments have a positive relationship with the overall methodological quality. Compared to non-Cochrane SRs, Cochrane SRs would usually fulfill rigorous editorial requirements of Cochrane Collaboration and adhere to strict methodological standards prior to publication [16]. These editorial procedures may serve as a gatekeeper to assure methodological quality of Cochrane SRs. There is a mixed pattern of methodological weakness across SRs led by corresponding authors from different regions. For example, SRs led by European corresponding authors performed better in assessing the potential impact of bias originating from primary studies, registering protocols *a priori*, and providing a list of excluded studies with justifications than those led by Asian corresponding authors. On the other hand, they performed worse in using a comprehensive literature search strategy than those led by American corresponding authors. Regardless of the corresponding author’s location, effort is still needed to improve the methodological quality of future SRs on AD treatments.

### The need for declaring conflicts of interest of the SR and reporting funding sources of primary studies

Unexpectedly, SRs of pharmacological treatments performed better than non-pharmacological interventions, especially in assessing the potential impact of bias originating from primary studies, conducting a comprehensive literature search, and providing a list of excluded studies with justifications. These observations may be related to the fact that SRs of pharmacological treatments have a higher chance of obtaining funding support from commercial sources. More resources may allow the team to perform the SR with more attention to methodological details. However, previous research showed that industry-sponsored SRs tend to be biased towards drawing a favorable conclusion [17]. Despite their relative strength in terms of rigor, evidence users should always pay attention to potential influence from funders when interpreting the results of SRs on pharmacological AD treatments.

In the current study, the majority of SRs on AD treatments performed well in reporting conflict of interest for the SR itself (98% of SRs rated “yes”). However, only 23.5% of the included SRs disclosed the funding sources of their primary studies. As industry sponsorship of treatment studies may lead to a more favorable result and biased conclusion [18], readers cannot judge whether financial interests might influence the conclusions of primary studies as well as the SR itself if funding sources of primary studies were not reported. Improvement towards the methodological quality of SRs on AD treatments can be made by declaring conflicts of interest and revealing funding sources of primary studies.

### Comparisons with similar studies

In this study, the proportion of SRs with high overall methodological quality (3.9%) is similar to that of SRs on osteoarthritis treatments (4.2%) [14], higher than SRs on osteoporosis treatments (1.0%) [15] or acupuncture (0.9%) [19], but lower than SRs on asthma treatments (8.8%) [13]. When considering the performance of individual AMSTAR2 critical items, our sample performs better in conducting a comprehensive literature search, using appropriate methods for meta-analysis, investigating publication bias, and discussing its potential impact, compared with SRs on osteoarthritis treatments, acupuncture, and asthma treatments. However, our sample is inferior to SR from these three clinical areas in terms of accounting for risk of bias in primary studies when interpreting results.

### Recommendations for future SRs

#### Registering systematic review protocols a priori

Registering SR protocols *a priori* can ensure transparency and minimize the influence of the reviewer’s bias caused by prior knowledge [20]. Protocol registration could be achieved by setting an a prior plan for the research question to be answered, establishing eligibility criteria for potential studies, and proposing methods for literature screening, quality appraising, and data synthesizing [20]. In this case, the proposed methods could also undergo peer evaluation, thus avoiding potential chance of duplication of research [21]. Since the launch of the International Prospective Register of Systematic Reviews (PROSPERO) in 2011 [22], there has been an increasing trend of registering SR protocols over the years [23, 24]. This trend may explain our findings, of which SRs published between 2019 and 2021 are more likely to register protocols a priori and justify deviations compared to those published between 2014 and 2015. That said, if we examine all included SRs of AD treatments, only 22.5% satisfy this criterion. Authors of future SRs on AD treatments are advised to register protocols a priori on open access electronic databases like PROSPERO or publish protocols in peer-reviewed journals.

#### Providing a list of excluded studies with justifications

Another approach to improve transparency and reproducibility, as well as to minimize the influence of reviewers on an SR is to provide a list of excluded studies with rationales for exclusion. This list helps inform readers of any completed primary studies that are most likely to be considered eligible but not included in the SR [25]. This may mitigate problems with restrictive eligibility criteria in specific SRs, allowing evidence users to retrieve relevant studies considered helpful in answering a particular clinical question. Documenting a list of excluded studies with reasons for exclusion is compulsory for Cochrane SRs but not for SRs published in most journals [16]. In this study, only 21.6% of SRs offered a list of excluded studies with rationales. It is recommended that SR authors, journal editors, and peer reviewers should be cautious about this issue.

#### Explaining study design selection

It is well established that RCT is the best primary study design for evaluating the effects of interventions [26]. Using randomization, RCT can prevent systematic baseline differences across different intervention groups by eliminating known and unknown confounders, and hence its results can quantify causal relationships with more confidence compared to observational designs [26]. When defining the eligibility criteria of an SR, study design selection should always be considered and justified in advance, even if it is restricted to RCTs. Such justification will reveal potential limitations caused by choice of study design. For example, when synthesizing evidence on treatment effects, exclusive inclusion of RCTs may lead to non-reporting of findings from observational studies. These observational studies may contain results on longer-term adverse effects, which are keys to decision-making, and readers should be alerted of such exclusion [27]. In our sample, only 6.9% justified the study design selection. SR authors may need to address this limitation in future works.

#### Strengths and limitations

Strengths of this study include (i) conducting a comprehensive database search to identify eligible SRs; (ii) assessing the methodological quality of an up-to-date sample of Cochrane and non-Cochrane SRs, covering both pharmacological treatments and non-pharmacological interventions; and (iii) adopting AMSRAR2, a validated tool to evaluate the overall methodological quality, as well as to assess detailed performance in each AMSTAR2 item so as to inform specific area for improvement.

One limitation is that methodological quality can only be assessed based on information reported in the published SR manuscripts. In this case, assessment results might be influenced by reporting the quality of the SRs and subsequently limiting the accuracy of our appraisal. Another restriction is that we only include SRs published in English. However, as a majority of SRs informing significant practice changes are published in English, our sample remains representative of SRs highly utilized internationally.

#### Implications

This study reveals that the majority of SRs on AD treatments are of low or critically low overall methodological quality. Flaws in the study design and conduct of SRs may cause underestimation or overestimation of the effectiveness of AD treatments, which may, in turn, mislead clinical practice if decisions are made based on biased findings. Therefore, to make informed policy and clinical decisions, policymakers, healthcare providers, and other evidence users should critically assess the methodological quality of SRs before the adoption of evidence. More importantly, SR authors, journal editors, and peer-reviewers are also suggested to use AMSTAR 2 and Cochrane Handbook for Systematic Reviews of Interventions as guidelines to ensure the scientific rigor of SRs prior to publication.

## Conclusions

The overall methodological quality of SRs on AD treatments is unsatisfactory, with only 3.9% appraised as having a high overall methodological quality. Cochrane SRs and SRs of pharmacological treatments have better overall quality. There is a critical need for improvements in the areas of protocol registration and publication, study design selection explanations, exclusion study details justifications, and primary study funding sources disclosure.

## Supplementary Information

**Additional file 1: Appendix 1.** Search strategies and results for systematic reviews on treatments of Alzheimer’s Disease. **Appendix 2.** Data extraction form for bibliographical characteristics. **Appendix 3.** AMSTAR 2 critical appraisal form & rating of overall methodological quality. **Appendix 4.** List of included study. **Appendix 5.** Methodological quality of each included systematic reviews (SRs) (*n* = 102). **Appendix 6a.** Associations between bibliographical characteristics of systematic reviews and individual AMSTAR-2 item performance - results of binary logistic regression. **Appendix 6b.** Associations between bibliographical characteristics of systematic reviews and individual AMSTAR-2 item performance - results of multinomial logistic regression.

## Data Availability

The data that support the findings of this study are available on request from the corresponding author.
