# Supplementary material for: Methodological quality of systematic reviews on treatments for Alzheimer’s disease: a cross-sectional study
Source: Alzheimers Res Ther. 2022 Oct 29;14:159. doi: 10.1186/s13195-022-01100-w (PMC9617345; doi:10.1186/s13195-022-01100-w)
Supplement: Supplementary file 1 — Additional file 1: Appendix 1. Search strategies and results for systematic reviews on treatments of Alzheimer’s Disease. Appendix 2. Data extraction form for bibliographical characteristics. Appendix 3. AMSTAR 2 critical appraisal form & rating of overall methodological quality. Appendix 4. List of included study. Appendix 5. Methodological quality of each included systematic reviews (SRs) (n = 102). Appendix 6a. Associations between bibliographical characteristics of systematic reviews and individual AMSTAR-2 item performance - results of binary logistic regression. Appendix 6b. Associations between bibliographical characteristics of systematic reviews and individual AMSTAR-2 item performance - results of multinomial logistic regression. [file 13195_2022_1100_MOESM1_ESM.docx]

# Appendix 1 Search strategies and results for systematic reviews on treatments of Alzheimer’s Disease

1. **Ovid MEDLINE(R) (1946 to February,2021)**

| **#** | **Search Statement** | **Results** |
| --- | --- | --- |
| 1 | exp Dementia/ | 171152 |
| 2 | Delirium, Dementia, Amnestic, Cognitive Disorders/ | 9328 |
| 3 | dement*.mp. | 118285 |
| 4 | alzheimer*.mp. | 143148 |
| 5 | ("organic brain disease" or "organic brain syndrome").mp. | 765 |
| 6 | "benign senescent forgetfulness".mp. | 18 |
| 7 | (cerebr* adj2 deteriorat*).mp. | 225 |
| 8 | (cerebral* adj2 insufficient*).mp. | 79 |
| 9 | 1 or 2 or 3 or 4 or 5 or 6 or 7 or 8 | 243582 |
| 10 | (MEDLINE or systematic review).tw. or meta analysis.pt. | 252094 |
| 11 | 9 and 10 | 4678 |
| 12 | limit 11 to (english language and humans and yr="2014 -Current") | 2828 |

1. **Embase (1910 to February,2021)**

| **#** | **Search Statement** | **Results** |
| --- | --- | --- |
| 1 | cognitive defect/ | 173443 |
| 2 | dement*.mp. | 216000 |
| 3 | alzheimer*.mp. | 255883 |
| 4 | ("organic brain disease" or "organic brain syndrome").mp. | 3342 |
| 5 | (cerebr* adj2 deteriorat*).mp. | 335 |
| 6 | (cerebral* adj2 insufficient*).mp. | 106 |
| 7 | Alzheimer disease/ | 209132 |
| 8 | AD.ab. | 209319 |
| 9 | 1 or 2 or 3 or 4 or 5 or 6 or 7 or 8 | 611115 |
| 10 | (meta-analysis or systematic review).tw. | 343720 |
| 11 | 9 and 10 | 10393 |
| 12 | limit 11 to (human and english language and yr="2014 -Current") | 7027 |

1. **PsycInfo (1806 to February Week 2 2021)**

| **#** | **Search Statement** | **Results** |
| --- | --- | --- |
| 1 | alzheimer*.mp. | 67410 |
| 2 | ("organic brain disease" or "organic brain syndrome").mp. | 866 |
| 3 | (cerebr* adj2 deteriorat*).mp. | 50 |
| 4 | (cerebral* adj2 insufficient*).mp. | 6 |
| 5 | Alzheimer's Disease/ | 48104 |
| 6 | AD.ab. | 50861 |
| 7 | 1 or 2 or 3 or 4 or 5 or 6 | 83566 |
| 8 | (meta-analysis or search:).tw. | 128324 |
| 9 | 7 and 8 | 3004 |
| 10 | limit 9 to (human and english language and yr="2014 -Current") | 1350 |

1. **Cochrane Database of Systematic Reviews (From database’s inception to Feb 2021)**

| **#** | **Search Statement** | **Results** |
| --- | --- | --- |
| #1 | MeSH descriptor: [Dementia] explode all trees | 6004 |
| #2 | MeSH descriptor: [Delirium] this term only | 702 |
| #3 | MeSH descriptor: [Wernicke Encephalopathy] this term only | 4 |
| #4 | MeSH descriptor: [Neurocognitive Disorders] this term only | 183 |
| #5 | dement* | 24324 |
| #6 | alzheimer* | 12641 |
| #7 | lewy* bod* | 51 |
| #8 | deliri* | 3988 |
| #9 | chronic cerebrovascular | 140 |
| #10 | organic brain disease or "organic brain syndrome" | 245 |
| #11 | normal pressure hydrocephalus and "shunt*" | 131 |
| #12 | benign senescent forgetfulness | 80 |
| #13 | cerebr* deteriorat* | 35 |
| #14 | cerebral* insufficient* | 30 |
| #15 | pick* disease | 123 |
| #16 | creutzfeldt or jcd or cjd | 291 |
| #17 | huntington* | 1163 |
| #18 | binswanger* | 108 |
| #19 | korsako* | 771 |
| #20 | (#1 OR #2 OR #3 OR #4 OR #5 OR #6 OR #7 OR #8 OR #9 OR #10 OR #11 OR #12 OR #13 OR #14 OR #15 OR #16 OR #17 OR #18 OR #19)  with Cochrane Library publication date Between Jan 2014 and Feb 2021, in Cochrane Reviews (Word variations have been searched) | 466 |

# Appendix 2 Data extraction form for bibliographical characteristics

| 1. Is this a Cochrane review? Yes / No |
| --- |
| 1. Is there an update of previous review? Yes / No |
| 1. Has harm been considered in the systematic review? Yes / No |
| 1. Did the authors of the systematic review search for English databases? Yes / No |
| 1. Did the authors of the systematic review search for non-English databases? Yes / No |
| 1. Is there any PRISMA-like flow diagram in the review? Yes / No |
| 1. Year of systematic review’s publication: _____ |
| 1. Impact factor of systematic review’s journal in the year before its publication: ____ |
| 1. Number of the review’s authors: _____ |
| 1. Number of ALL included studies (regardless whether they have been included in meta-analysis or not):___ |
| 1. Number of participants in ALL included studies: _____ |
| 1. Location of the corresponding author: _____   a. Europe b. America c. Asia d. Oceania e. Africa f. Not reported |
| 1. Funding location of the systematic review: _____   a. Europe b. America c. Asia d. Oceania e. Africa f. Not reported g. not applicable |
| 1. Type of the treatment/interventions   a. Non-pharmacological b. Pharmacological c. Both types |
| 1. Was “year of coverage” reported?   a. Yes (reported both starting and ending years) b. Partially (only reported starting years or ending years)  c. Not mentioned |
| 1. Searching terms reported   a. Not research term  b. Topics/free text/keywords/MeSH  c. Full Boolean  d. Readers are referred elsewhere for full search strategy |
| 1. This systematic review included primary studies of the following languages:   a. English only b. Language other than English c. English and language other than English  d. Language criteria not reported |
| 1. Quality of the primary studies is assessed by which tools?   a. Cochrane risk of bias  b. Jadad scale  c. Schulz approach  d. Effective Public Health Practice Project Quality Assessment Tool (EPHPP)  e. Juni  f. Chalmer scale  g. Pedro Scale  h. Delphi list  i. More than one tools  j. Others |

Notes: PRISMA, Preferred Reporting Items for Systematic Reviews and Meta-Analyses; MeSH, Medical Subject Headings; RCT, randomised controlled trial; RoB, Risk of bias

# Appendix 3 AMSTAR 2 critical appraisal form & rating of overall methodological quality

| 1. **Did the research questions and inclusion criteria for the review include the components of PICO?**  \| For Yes  🞏 Population  🞏 Intervention  🞏 Comparator group  🞏 Outcome \| Optional (recommended)  🞏 Timeframe for follow-up \| **🞏 Yes**  **🞏 No** \| \| --- \| --- \| --- \| |
| --- | --- | --- | --- |
| 1. ***Did the report of the review contain an explicit statement that the review methods were established prior to the conduct of the review and did the report justify any significant deviations from the protocol?**  \| For Partial Yes:  The authors state that they had a written protocol or guide that included ALL the following:  🞏 review question(s)  🞏 a search strategy  🞏 inclusion/exclusion criteria  🞏 a RoB assessment \| For Yes:  As for partial yes, plus the protocol should be registered and should also have specified:  🞏 a meta-analysis/synthesis  plan, if appropriate, and  🞏 a plan for investigating  causes of heterogeneity  🞏 justification for any  deviations from the protocol \| **🞏 Yes**  **🞏 Partial Yes**  **🞏 No** \| \| --- \| --- \| --- \| |
| 1. **Did the review authors explain their selection of the study designs for inclusion in the review?**  \| For Yes, the review should satisfy ONE of the following:  🞏 Explanation for including only RCTs  🞏 OR Explanation for including only NRSI  🞏 OR Explanation for including both RCTs and NRSI \| **🞏 Yes**  **🞏 No** \| \| --- \| --- \| |
| 1. ***Did the review authors use a comprehensive literature search strategy?**  \| For Partial Yes (all the following):  🞏 searched at least 2 databases (relevant to research question)  🞏 provided key word and/or search strategy  🞏 justified publication restrictions (eg, language) \| For Yes, should also have (all the following):  🞏 searched the reference lists/bibliographies of included studies  🞏 searched trial/study registries  🞏 included/consulted content experts in the field  🞏 where relevant, searched for grey literature  🞏 conducted search within 24 months of completion of the review \| **🞏 Yes**  **🞏 Partial Yes**  **🞏 No** \| \| --- \| --- \| --- \| |
| 1. **Did the review authors perform study selection in duplicate?**  \| For Yes, either ONE of the following:  🞏 at least two reviewers independently agreed on selection of eligible studies and achieved consensus on which studies to include  🞏 OR two reviewers selected a sample of eligible studies and achieved good agreement (at least 80 per cent), with the remainder selected by one reviewer \| **🞏 Yes**  **🞏 No** \| \| --- \| --- \| |
| 1. **Did the review authors perform data extraction in duplicate?**  \| For Yes, either ONE of the following:  🞏 at least two reviewers achieved consensus on which data to extract from included studies  🞏 OR two reviewers extracted data from a sample of eligible studies and achieved good agreement (at least 80 per cent), with the remainder extracted by one reviewer \| **🞏 Yes**  **🞏 No** \| \| --- \| --- \| |
| 1. ***Did the review authors provide a list of excluded studies and justify the exclusions?**  \| For Partial Yes:  🞏 provided a list of all potentially relevant studies that were read in full text form but excluded from the review \| For Yes, must also have:  🞏 Justified the exclusion from the review of each potentially relevant study \| **🞏 Yes**  **🞏 Partial Yes**  **🞏 No** \| \| --- \| --- \| --- \| |
| 1. **Did the review authors describe the included studies in adequate detail?**  \| For Partial Yes (ALL the following):  🞏 described populations  🞏 described interventions  🞏 described comparators  🞏 described outcomes  🞏 described research designs \| For Yes, should also have ALL the following:  🞏 described populations in detail  🞏 described intervention and comparator in detail (including does where relevant)  🞏 described study’s setting  🞏 timeframe for follow-up \| **🞏 Yes**  **🞏 Partial Yes**  **🞏 No** \| \| --- \| --- \| --- \| |
| 1. ***Did the review authors use a satisfactory technique for assessing the RoB in individual studies that were included in the review?**  \| **RCTs**  For Partial Yes, must have assessed RoB from  🞏 unconcealed allocation, *and*  🞏 lack of blinding of patients and assessors when assessing outcomes (unnecessary for objective outcomes such as all cause mortality) \| For Yes, must also have assessed RoB from:  🞏 allocation sequence that was not truly random, *and*  🞏 selection of the reported result from among multiple measurements or analyses of a specified outcome \| **🞏 Yes**  **🞏 Partial Yes**  **🞏 No**  **🞏 Includes only NRSI** \| \| --- \| --- \| --- \| \| **NRSI**  For Partial Yes, must have assessed RoB:  🞏 from confounding, *and*  🞏 from selection bias \| For Yes, must also have assessed RoB:  🞏 methods used to ascertain exposures and outcomes, *and*  🞏 selection o the reported result from among multiple measurements or analyses of a specified outcome \| **🞏 Yes**  **🞏 Partial Yes**  **🞏 No**  **🞏 Includes only RCTs** \| |
| 1. **Did the review authors report on the sources of funding for the studies included in the review?**  \| For Yes  🞏 Must have reported on the sources of funding for individual studies included in the review. Note: Reporting that the reviewers looked for this information but it was not reported by study authors also qualifies \| **🞏 Yes**  **🞏 No** \| \| --- \| --- \| |
| 1. ***If meta-analysis was performed did the review authors use appropriate methods for statistical combination of results?**  \| **RCTs**  For Yes:  🞏 The authors justified combining the data in a meta-analysis  🞏 AND they used an appropriate weighted technique to combine study results and adjusted for heterogeneity if present  🞏 AND investigated the causes of any heterogeneity \| **🞏 Yes**  **🞏 No**  **🞏 No meta-analysis conducted** \| \| --- \| --- \| \| **For NRSI**  For Yes  🞏 The authors justified combining the data in a meta-analysis  🞏 AND they used an appropriate weighted technique to combine study results, adjusting for heterogeneity if present  🞏 AND they statistically combined effect estimates from NRSI that were adjusted for confounding, rather than combining raw data, or justified combining raw data when adjusted effect estimates were not available  🞏 AND they reported separate summary estimates for RCTs and NRSI separately when both were included in the review \| **🞏 Yes**  **🞏 No**  **🞏 No meta-analysis conducted** \| |
| 1. **If meta-analysis was performed, did the review authors assess the potential impact of RoB in individual studies on the results of the meta-analysis or other evidence synthesis?**  \| For Yes  🞏 included only low RoB RCTs  🞏 OR, if the pooled estimate was based on RCTs and/or NRSI at variable RoB, the authors performed analyses to investigate possible impact of RoB on summary estimates of effect \| **🞏 Yes**  **🞏 No**  **🞏 No meta-analysis conducted** \| \| --- \| --- \| |
| 1. ***Did the review authors account for RoB in individual studies when interpreting/discussing the results of the review?**  \| For Yes:  🞏 included only low RoB RCTs  🞏 OR, if RCTs with moderate or high RoB, or NRSI were included the review provided a discussion of the likely impact of RoB on the results \| **🞏 Yes**  **🞏 No** \| \| --- \| --- \| |
| 1. **Did the review authors provide a satisfactory explanation for, and discussion of, any heterogeneity observed in the results of the review?**  \| For Yes:  🞏 There was no significant heterogeneity in the results  🞏 OR if heterogeneity was present the authors performed an investigation of sources of any heterogeneity in the results and discussed the impact of this on the results of the review \| **🞏 Yes**  **🞏 No** \| \| --- \| --- \| |
| 1. ***If they performed quantitative synthesis did the review authors carry out an adequate investigation of publication bias (small study bias) and discuss its likely impact on the results of the review?**  \| For Yes:  🞏 performed graphical or statistical tests for publication bias and discussed the likelihood and magnitude of impact of publication bias \| **🞏 Yes**  **🞏 No**  **🞏 No meta-analysis conducted** \| \| --- \| --- \| |
| 1. **Did the review authors report any potential sources of conflict of interest, including any funding they received for conducting the review?**  \| For Yes:  🞏 The authors reported no competing interests OR  🞏 The authors described their funding sources and how they managed potential conflicts of interest \| **🞏 Yes**  **🞏 No** \| \| --- \| --- \|   **Rating of overall methodological quality**  **Critical items(marked with *): 2, 4, 7, 9, 11, 13, 15;**  **Non-critical items: 1,3, 5, 6, 8, 10, 12, 14, 16.**  **High**  No or one non-critical weakness: the systematic review provides an accurate and comprehensive summary of the results of the available studies that address the question of interest  **Moderate**  More than one non-critical weakness*: the systematic review has more than one weakness but no critical flaws. It may provide an accurate summary of the results of the available studies that were included in the review  **Low**  One critical flaw with or without non-critical weaknesses: the review has a critical flaw and may not provide an accurate and comprehensive summary of the available studies that address the question of interest  **Critically low**  More than one critical flaw with or without non-critical weaknesses: the review has more than one critical flaw and should not be relied on to provide an accurate and comprehensive summary of the available studies  *Multiple non-critical weaknesses may diminish confidence in the review and it may be appropriate to move the overall appraisal down from moderate to low confidence |

RCT, randomised controlled trial; NRSI, non-randomised studies of interventions; RoB, risk of bias

*Reference: Shea BJ, Reeves BC, Wells G, Thuku M, Hamel C, Moran J, et al. AMSTAR 2: a critical appraisal tool for systematic reviews that include randomised or non-randomised studies of healthcare interventions, or both. BMJ. 2017;358:j4008.*

# Appendix 4 List of included study

1. Abushouk, A. I., et al. (2017). "Bapineuzumab for mild to moderate Alzheimer's disease: a meta-analysis of randomized controlled trials." BMC Neurol 17(1): 66.

2. Ayati, Z., et al. (2020). "Saffron for mild cognitive impairment and dementia: a systematic review and meta-analysis of randomised clinical trials." BMC Complement Med Ther 20(1): 333.

3. Birks, J. S. and J. Grimley Evans (2015). "Rivastigmine for Alzheimer's disease." Cochrane Database Syst Rev(4): CD001191.

4. Birks, J. S. and R. J. Harvey (2018). "Donepezil for dementia due to Alzheimer's disease." Cochrane Database Syst Rev 6(6): CD001190.

5. Blanco-Silvente, L., et al. (2019). "Study of the strength of the evidence and the redundancy of the research on pharmacological treatment for Alzheimer's disease: a cumulative meta-analysis and trial sequential analysis." Eur J Clin Pharmacol 75(12): 1659-1667.

6. Burckhardt, M., et al. (2016). "Omega-3 fatty acids for the treatment of dementia." Cochrane Database Syst Rev 4: CD009002.

7. Burckhardt, M., et al. (2020). "Souvenaid for Alzheimer's disease." Cochrane Database Syst Rev 12(12): CD011679.

8. Cai, M., et al. (2019). "Transcranial Direct Current Stimulation Improves Cognitive Function in Mild to Moderate Alzheimer Disease: A Meta-Analysis." Alzheimer Dis Assoc Disord 33(2): 170-178.

9. Chau, S., et al. (2015). "Latrepirdine for Alzheimer's disease." Cochrane Database Syst Rev(4): CD009524.

10. Chen, J., et al. (2019). "Different durations of cognitive stimulation therapy for Alzheimer's disease: a systematic review and meta-analysis." Clin Interv Aging 14: 1243-1254.

11. Chen, R., et al. (2017). "Treatment effects between monotherapy of donepezil versus combination with memantine for Alzheimer disease: A meta-analysis." PLoS One 12(8): e0183586.

12. Cheng, H., et al. (2016). "The peroxisome proliferators activated receptor-gamma agonists as therapeutics for the treatment of Alzheimer's disease and mild-to-moderate Alzheimer's disease: a meta-analysis." Int J Neurosci 126(4): 299-307.

13. Chou, Y. H., et al. (2020). "A systematic review and meta-analysis of rTMS effects on cognitive enhancement in mild cognitive impairment and Alzheimer's disease." Neurobiol Aging 86: 1-10.

14. Dauwan, M., et al. (2019). "Physical exercise improves quality of life, depressive symptoms, and cognition across chronic brain disorders: a transdiagnostic systematic review and meta-analysis of randomized controlled trials." J Neurol.

15. de Almeida, S. I. L., et al. (2020). "Home-Based Physical Activity Programs for People With Dementia: Systematic Review and Meta-Analysis." Gerontologist 60(8): 600-608.

16. Den, H., et al. (2020). "Efficacy of probiotics on cognition, and biomarkers of inflammation and oxidative stress in adults with Alzheimer's disease or mild cognitive impairment - a meta-analysis of randomized controlled trials." Aging (Albany NY) 12(4): 4010-4039.

17. Dominik, G., et al. (2019). "Acetylcholinesterase inhibitors combined with memantine for moderate to severe Alzheimer's disease: A meta-analysis." Swiss Medical Weekly 149(25-26).

18. Dong, X., et al. (2018). "Repetitive transcranial magnetic stimulation for the treatment of Alzheimer's disease: A systematic review and meta-analysis of randomized controlled trials." PLoS One 13(10): e0205704.

19. Du, Z., et al. (2018). "Physical activity can improve cognition in patients with alzheimer's disease: A systematic review and meta-analysis of randomized controlled trials." Clinical Interventions in Aging 13: 1593-1603.

20. Farina, N., et al. (2014). "The effect of exercise interventions on cognitive outcome in Alzheimer's disease: A systematic review." International Psychogeriatrics 26(1): 9-18.

21. Feustel, A. C., et al. (2020). "Risks and benefits of unapproved disease-modifying treatments for neurodegenerative disease." Neurology 94(1): e1-e14.

22. Gauthier, S., et al. (2015). "Cerebrolysin in mild-to-moderate Alzheimer's disease: A meta-analysis of randomized controlled clinical trials." Dementia and Geriatric Cognitive Disorders 39(5-6): 332-347.

23. Gupta, P. P., et al. (2015). "Role of traditional nonsteroidal anti-inflammatory drugs in Alzheimer's disease: A meta-analysis of randomized clinical trials." American Journal of Alzheimer's Disease and Other Dementias 30(2): 178-182.

24. Hsu, W. Y., et al. (2015). "Effects of noninvasive brain stimulation on cognitive function in healthy aging and Alzheimer's disease: a systematic review and meta-analysis." Neurobiol Aging 36(8): 2348-2359.

25. Huang, Q., et al. (2019). "Effectiveness of Acupuncture for Alzheimer's Disease: An Updated Systematic Review and Meta-analysis." Curr Med Sci 39(3): 500-511.

26. Iketani, R., et al. (2018). "Apolipoprotein E Gene Polymorphisms Affect the Efficacy of Thiazolidinediones for Alzheimer's Disease: A Systematic Review and Meta-Analysis." Biol Pharm Bull 41(7): 1017-1023.

27. Jia, R. X., et al. (2019). "Effects of physical activity and exercise on the cognitive function of patients with Alzheimer disease: a meta-analysis." BMC Geriatr 19(1): 181.

28. Jiang, D., et al. (2015). "Efficacy and safety of galantamine treatment for patients with Alzheimer's disease: A meta-analysis of randomized controlled trials." Journal of Neural Transmission 122(8): 1157-1166.

29. Jiang, J. and H. Jiang (2015). "Efficacy and adverse effects of memantine treatment for Alzheimer's disease from randomized controlled trials." Neurol Sci 36(9): 1633-1641.

30. Kim, K., et al. (2017). "Cognitive Stimulation as a Therapeutic Modality for Dementia: A Meta-Analysis." Psychiatry Investigation 14(5): 626-639.

31. Kim, Y. and S. H. Cho (2020). "Danggui-Shaoyao-San for dementia: A PRISMA-compliant systematic review and meta-analysis." Medicine (Baltimore) 99(4): e18507.

32. Kishi, T., et al. (2015). "Protection against brain atrophy by anti-dementia medication in mild cognitive impairment and Alzheimer's disease: Meta-analysis of longitudinal randomized placebo-controlled trials." International Journal of Neuropsychopharmacology 18(12): 1-7.

33. Kishi, T., et al. (2017a). "The effects of memantine on behavioral disturbances in patients with Alzheimer's disease: a meta-analysis." Neuropsychiatr Dis Treat 13: 1909-1928.

34. Kishi, T., et al. (2017b). "Memantine for Alzheimer's Disease: An Updated Systematic Review and Meta-analysis." J Alzheimers Dis 60(2): 401-425.

35. Kishi, T., et al. (2018). "Memantine treatment for Japanese patients with moderate to severe Alzheimer's disease: a meta-analysis of double-blind, randomized, placebo-controlled trials." Neuropsychiatr Dis Treat 14: 2915-2922.

36. Kishi, T., et al. (2020). "Efficacy and Safety of Psychostimulants for Alzheimer's Disease: A Systematic Review and Meta-Analysis." Pharmacopsychiatry 53(3): 109-114.

37. Kruger, J. F., et al. (2021). "Probiotics for dementia: a systematic review and meta-analysis of randomized controlled trials." Nutr Rev 79(2): 160-170.

38. Kubo, M., et al. (2015). "Histamine H3 receptor antagonists for Alzheimer's disease: A systematic review and meta-analysis of randomized placebo-controlled trials." Journal of Alzheimer's Disease 48(3): 667-671.

39. Lan, J., et al. (2020). "Acupuncture for cognitive impairment in vascular dementia, alzheimer's disease and mild cognitive impairment: A systematic review and meta-analysis." European Journal of Integrative Medicine 35 (no pagination).

40. Li, C., et al. (2016). "Amyloid beta directed antibody for Alzheimer's disease, an evidence based meta-analysis." Cell Mol Biol (Noisy-le-grand) 62(4): 83-87.

41. Li, D. D., et al. (2019). "Meta-Analysis of Randomized Controlled Trials on the Efficacy and Safety of Donepezil, Galantamine, Rivastigmine, and Memantine for the Treatment of Alzheimer's Disease." Front Neurosci 13(MAY): 472.

42. Li, M. M., et al. (2014). "Efficacy of vitamins B supplementation on mild cognitive impairment and Alzheimer's disease: a systematic review and meta-analysis." Curr Alzheimer Res 11(9): 844-852.

43. Liang, T., et al. (2015). "Statins for Treating Alzheimer's Disease: Truly Ineffective?" Eur Neurol 73(5-6): 360-366.

44. Liao, X., et al. (2015). "Repetitive transcranial magnetic stimulation as an alternative therapy for cognitive impairment in Alzheimer's disease: A meta-analysis." Journal of Alzheimer's Disease 48(2): 463-472.

45. Liao, Z., et al. (2020). "Meta-analysis of Ginkgo biloba Preparation for the Treatment of Alzheimer's Disease." Clin Neuropharmacol 43(4): 93-99.

46. Lin, Y., et al. (2019). "The role of repetitive transcranial magnetic stimulation (rTMS) in the treatment of cognitive impairment in patients with Alzheimer's disease: A systematic review and meta-analysis." J Neurol Sci 398: 184-191.

47. Liu, J. and L.-N. Wang (2019). "Intravenous immunoglobulins for Alzheimer's disease and mild cognitive impairment due to Alzheimer's disease: A systematic review with meta-analysis." Expert Review of Neurotherapeutics 19(6): 475-480.

48. Liu, J., et al. (2015). "Peroxisome proliferator-activated receptor-gamma agonists for Alzheimer's disease and amnestic mild cognitive impairment: a systematic review and meta-analysis." Drugs Aging 32(1): 57-65.

49. Lu, L., et al. (2020). "Anti-Abeta agents for mild to moderate Alzheimer's disease: systematic review and meta-analysis." J Neurol Neurosurg Psychiatry 91(12): 1316-1324.

50. Ma, H. K., et al. (2018). "Chinese Medicine for Alzheimer's Disease: A Meta-Analysis of Randomized Controlled Trials." Chin J Integr Med 24(12): 938-943.

51. Ma, H., et al. (2014). "The efficacy and safety of atypical antipsychotics for the treatment of dementia: a meta-analysis of randomized placebo-controlled trials." J Alzheimers Dis 42(3): 915-937.

52. Manolopoulos, A., et al. (2019). "Intravenous Immunoglobulin for Patients With Alzheimer's Disease: A Systematic Review and Meta-Analysis." Am J Alzheimers Dis Other Demen 34(5): 281-289.

53. Matsunaga, S., et al. (2015a). "Lithium as a treatment for Alzheimer's disease: A systematic review and meta-analysis." Journal of Alzheimer's Disease 48(2): 403-410.

54. Matsunaga, S., et al. (2015b). "Combination therapy with cholinesterase inhibitors and memantine for Alzheimer's disease: A systematic review and meta-analysis." International Journal of Neuropsychopharmacology 18(5): 1-11.

55. Matsunaga, S., et al. (2015c). "Memantine monotherapy for Alzheimer's disease: a systematic review and meta-analysis." PLoS One 10(4): e0123289.

56. Matsunaga, S., et al. (2016). "Yokukansan in the treatment of behavioral and psychological symptoms of dementia: An updated meta-analysis of randomized controlled trials." Journal of Alzheimer's Disease 54(2): 635-643.

57. Matsunaga, S., et al. (2019a). "Efficacy and Safety of Glycogen Synthase Kinase 3 Inhibitors for Alzheimer's Disease: A Systematic Review and Meta-Analysis." J Alzheimers Dis 69(4): 1031-1039.

58. Matsunaga, S., et al. (2019b). "Efficacy and safety of idalopirdine for Alzheimer's disease: a systematic review and meta-analysis." Int Psychogeriatr 31(11): 1627-1633.

59. May, B. H., et al. (2018). "Comparisons between traditional medicines and pharmacotherapies for Alzheimer disease: A systematic review and meta-analysis of cognitive outcomes." International Journal of Geriatric Psychiatry 33(3): 449-458.

60. McCleery, J. and A. L. Sharpley (2020). "Pharmacotherapies for sleep disturbances in dementia." Cochrane Database Syst Rev 11(11): CD009178.

61. McGuinness, B., et al. (2014). "Statins for the treatment of dementia." Cochrane Database of Systematic Reviews(7).

62. McShane, R., et al. (2019). "Memantine for dementia." Cochrane Database Syst Rev 3(3): CD003154.

63. Miguel-Alvarez, M., et al. (2015). "Non-steroidal anti-inflammatory drugs as a treatment for Alzheimer's disease: A systematic review and meta-analysis of treatment effect." Drugs & Aging 32(2): 139-147.

64. Okuya, M., et al. (2018). "Efficacy, Acceptability, and Safety of Intravenous Immunoglobulin Administration for Mild-To-Moderate Alzheimer's Disease: A Systematic Review and Meta-Analysis." Journal of Alzheimer's Disease 66(4): 1379-1387.

65. Onakpoya, I. J. and C. J. Heneghan (2017). "The efficacy of supplementation with the novel medical food, Souvenaid, in patients with Alzheimer's disease: A systematic review and meta-analysis of randomized clinical trials." Nutr Neurosci 20(4): 219-227.

66. O'Regan, J., et al. (2015). "Cholinesterase inhibitor discontinuation in patients with Alzheimer's disease: A meta-analysis of randomized controlled trials." The Journal of Clinical Psychiatry 76(11): e1424-e1431.

67. Orgeta, V., et al. (2017). "Efficacy of Antidepressants for Depression in Alzheimer's Disease: Systematic Review and Meta-Analysis." J Alzheimers Dis 58(3): 725-733.

68. Penninkilampi, R., et al. (2017). "Safety and Efficacy of Anti-Amyloid-beta Immunotherapy in Alzheimer's Disease: A Systematic Review and Meta-Analysis." J Neuroimmune Pharmacol 12(1): 194-203.

69. Rao, A. K., et al. (2014). "Systematic review of the effects of exercise on activities of daily living in people with Alzheimer's disease." Am J Occup Ther 68(1): 50-56.

70. Ruthirakuhan, M. T., et al. (2018). "Pharmacological interventions for apathy in Alzheimer's disease." Cochrane Database of Systematic Reviews(5).

71. Ruthirakuhan, M., et al. (2019). "Natural and Synthetic Cannabinoids for Agitation and Aggression in Alzheimer's Disease: A Meta-Analysis." J Clin Psychiatry 80(2): 29.

72. Sampson, E. L., et al. (2014). "Metal protein attenuating compounds for the treatment of Alzheimer's dementia." Cochrane Database of Systematic Reviews(2).

73. Schmidt, R., et al. (2015). "EFNS-ENS/EAN Guideline on concomitant use of cholinesterase inhibitors and memantine in moderate to severe Alzheimer's disease." Eur J Neurol 22(6): 889-898.

74. Sepehry, A. A., et al. (2017). "Pharmacological Therapy for Apathy in Alzheimer's Disease: A Systematic Review and Meta-Analysis." Can J Neurol Sci 44(3): 267-275.

75. Strohle, A., et al. (2015). "Drug and exercise treatment of Alzheimer disease and mild cognitive impairment: A systematic review and meta-analysis of effects on cognition in randomized controlled trials." The American Journal of Geriatric Psychiatry 23(12): 1234-1249.

76. Su, J., et al. (2015). "Long-term effectiveness of rivastigmine patch or capsule for mild-to-severe Alzheimer's disease: A meta-analysis." Expert Review of Neurotherapeutics 15(9): 1093-1103.

77. Tan, C. C., et al. (2014). "Efficacy and safety of donepezil, galantamine, rivastigmine, and memantine for the treatment of Alzheimer's disease: a systematic review and meta-analysis." J Alzheimers Dis 41(2): 615-631.

78. Tan, L., et al. (2015a). "Efficacy and safety of atypical antipsychotic drug treatment for dementia: A systematic review and meta-analysis." Alzheimer's Research and Therapy 7(1).

79. Tan, M. S., et al. (2015b). "Efficacy and adverse effects of ginkgo biloba for cognitive impairment and dementia: a systematic review and meta-analysis." J Alzheimers Dis 43(2): 589-603.

80. Tsoi, K. K. F., et al. (2016). "Combination Therapy Showed Limited Superiority Over Monotherapy for Alzheimer Disease: A Meta-analysis of 14 Randomized Trials." Journal of the American Medical Directors Association 17(9): 863.e861-863.e868.

81. Wang, J., et al. (2015). "Pharmacological treatment of neuropsychiatric symptoms in Alzheimer's disease: a systematic review and meta-analysis." J Neurol Neurosurg Psychiatry 86(1): 101-109.

82. Wang, X., et al. (2020a). "Repetitive transcranial magnetic stimulation for cognitive impairment in Alzheimer's disease: a meta-analysis of randomized controlled trials." J Neurol 267(3): 791-801.

83. Wang, X., et al. (2020b). "The role of noninvasive brain stimulation for behavioral and psychological symptoms of dementia: a systematic review and meta-analysis." Neurol Sci 41(5): 1063-1074.

84. Wang, Y. Y., et al. (2017). "Meta-analysis of randomized, double-blind, placebo-controlled trials of melatonin in Alzheimer's disease." Int J Geriatr Psychiatry 32(1): 50-57.

85. Wang, Y., et al. (2020c). "A meta-analysis of the effect of music therapy on Alzheimer's disease." International Journal of Clinical and Experimental Medicine 13(2): 317-329.

86. Wang, Y. Y., et al. (2020d). "Effectiveness and Safety of Acupuncture for the Treatment of Alzheimer's Disease: A Systematic Review and Meta-Analysis." Front Aging Neurosci 12: 98.

87. Wang, Y., et al. (2016). "Ginseng for Alzheimer's Disease: A Systematic Review and Meta-Analysis of Randomized Controlled Trials." Curr Top Med Chem 16(5): 529-536.

88. Wei, S., et al. (2020). "The effect of nonpharmacologic therapy on global cognitive functions in patients with Alzheimer's disease: an updated meta-analysis of randomized controlled trials." Int J Neurosci 130(1): 28-44.

89. Xing, S. H., et al. (2014). "Huperzine A in the treatment of alzheimer's disease and vascular dementia: A meta-analysis." Evidence-based Complementary and Alternative Medicine 2014 (no pagination).

90. Xu, J., et al. (2015). "Melatonin for sleep disorders and cognition in dementia: a meta-analysis of randomized controlled trials." Am J Alzheimers Dis Other Demen 30(5): 439-447.

91. Xuan, K., et al. (2020). "The efficacy of statins in the treatment of Alzheimer's disease: a meta-analysis of randomized controlled trial." Neurol Sci 41(6): 1391-1404.

92. Yang, G., et al. (2016). "Ginkgo Biloba for Mild Cognitive Impairment and Alzheimer's Disease: A Systematic Review and Meta-Analysis of Randomized Controlled Trials." Curr Top Med Chem 16(5): 520-528.

93. Yang, M., et al. (2014). "A systematic review on natural medicines for the prevention and treatment of Alzheimer's disease with meta-analyses of intervention effect of ginkgo." Am J Chin Med 42(3): 505-521.

94. Zeng, L. F., et al. (2015). "Oral Chinese herbal medicine for kidney nourishment in Alzheimer's disease: a systematic review of the effect on MMSE index measures and safety." Complement Ther Med 23(2): 283-297.

95. Zhang, W., et al. (2016a). "Exogenous melatonin for sleep disorders in neurodegenerative diseases: a meta-analysis of randomized clinical trials." Neurol Sci 37(1): 57-65.

96. Zhang, X., et al. (2016b). "Efficacy of galantamine in treatment of Alzheimer's disease: An update meta-analysis." International Journal of Clinical and Experimental Medicine 9(4): 7423-7430.

97. Zhang, Y., et al. (2019). "Chinese herbal medicines on cognitive function and activity of daily living in senior adults with Alzheimer's disease: a systematic review and meta-analysis." Integr Med Res 8(2): 92-100.

98. Zhou, C., et al. (2020). "The Effect of Hormone Replacement Therapy on Cognitive Function in Female Patients With Alzheimer's Disease: A Meta-Analysis." Am J Alzheimers Dis Other Demen 35: 1533317520938585.

99. Zhou, J., et al. (2015). "The effectiveness and safety of acupuncture for patients with Alzheimer disease: a systematic review and meta-analysis of randomized controlled trials." Medicine (Baltimore) 94(22): e933.

100. Zhou, S., et al. (2017). "Acupuncture plus Herbal Medicine for Alzheimer's Disease: A Systematic Review and Meta-Analysis." Am J Chin Med 45(7): 1327-1344.

101. Zhu, L. N., et al. (2019). "Curcumin intervention for cognitive function in different types of people: A systematic review and meta-analysis." Phytother Res 33(3): 524-533.

102. Zhu, X. C., et al. (2015). "Physiotherapy intervention in Alzheimer's disease: systematic review and meta-analysis." J Alzheimers Dis 44(1): 163-174.

# Appendix 5 Methodological quality of each included systematic reviews (SRs) (n=102)

| SRs | Item 1 | Item 2* | Item 3 | Item 4* | Item 5 | Item 6 | Item 7* | Item 8 | Item 9* | Item 10 | Item 11* | Item 12 | Item 13* | Item 14 | Item 15* | Item 16 | Overall |
| --- | --- | --- | --- | --- | --- | --- | --- | --- | --- | --- | --- | --- | --- | --- | --- | --- | --- |
| Abushouk 2017 | Y | 1 | PY | N | Y | Y | Y | Y | Y | Y | N | Y | Y | Y | N | Y | Y |
| Ayati 2020 | Y | 1 | Y | N | PY | Y | N | N | Y | Y | N | Y | Y | Y | N | Y | Y |
| Birks 2015 | Y | 1 | Y | N | Y | N | N | Y | Y | Y | Y | Y | Y | Y | Y | Y | Y |
| Birks 2018 | Y | 1 | Y | Y | Y | Y | N | Y | Y | Y | Y | Y | Y | Y | Y | N | Y |
| Blanco-Silvente 2019 | Y | 1 | Y | N | PY | N | Y | N | Y | Y | N | Y | Y | Y | N | Y | Y |
| Burckhardt 2016 | Y | 1 | Y | Y | Y | Y | Y | Y | Y | Y | Y | Y | Y | Y | Y | Y | Y |
| Burckhardt 2020 | Y | 1 | Y | N | Y | Y | Y | Y | Y | Y | Y | Y | Y | Y | Y | Y | Y |
| Cai 2019 | Y | 1 | PY | N | PY | N | Y | N | Y | Y | N | Y | N | N | Y | Y | Y |
| Chau 2015 | Y | 0 | Y | N | Y | Y | Y | N | Y | Y | Y | N | N | Y | Y | Y | Y |
| Chen 2017 | Y | 1 | PY | N | PY | N | Y | N | Y | Y | N | Y | N | N | Y | Y | Y |
| Chen 2019 | Y | 1 | PY | N | PY | Y | Y | N | Y | Y | N | Y | N | Y | N | N | Y |
| Cheng 2016 | Y | 1 | PY | N | PY | N | Y | N | Y | Y | N | Y | Y | Y | Y | Y | Y |
| Chou 2020 | Y | 1 | PY | N | PY | N | Y | Y | Y | Y | N | Y | N | N | Y | Y | Y |
| Dauwan 2019 | Y | 1 | PY | N | PY | Y | N | N | Y | Y | N | Y | Y | Y | Y | Y | Y |
| de Almeida 2020 | Y | 0 | Y | N | PY | Y | N | N | Y | Y | N | N | Y | Y | Y | Y | Y |
| Den 2020 | Y | 1 | Y | N | Y | Y | Y | Y | Y | Y | N | Y | N | Y | Y | Y | Y |
| Dominik 2019 | Y | 1 | Y | N | PY | Y | N | N | Y | Y | Y | Y | N | Y | Y | Y | Y |
| Dong 2018 | Y | 1 | Y | N | PY | Y | Y | N | Y | Y | N | Y | N | Y | Y | Y | Y |
| Du 2018 | Y | 1 | PY | Y | PY | Y | Y | N | Y | Y | N | Y | N | N | Y | Y | Y |
| Farina 2014 | Y | 1 | PY | N | PY | N | N | N | Y | Y | N | Y | N | N | Y | Y | Y |
| Feustel 2020 | Y | 1 | Y | N | Y | N | N | N | Y | Y | N | Y | N | N | Y | N | Y |
| Gauthier 2015 | Y | 1 | PY | N | N | N | N | N | Y | Y | N | Y | Y | Y | Y | N | Y |
| Gupta 2015 | Y | 1 | N | N | PY | N | N | N | Y | N | N | Y | N | N | N | Y | Y |
| Hsu 2015 | Y | 1 | PY | N | PY | N | N | N | Y | Y | N | Y | N | Y | Y | Y | Y |
| Huang 2019 | Y | 1 | PY | N | Y | N | Y | N | Y | Y | N | Y | N | Y | Y | Y | Y |
| Iketani 2018 | Y | 1 | PY | N | Y | Y | Y | N | Y | Y | N | Y | Y | Y | Y | Y | Y |
| Jia 2019 | Y | 1 | PY | N | PY | Y | Y | N | Y | Y | N | Y | N | Y | Y | Y | Y |
| Jiang 2015 | Y | 0 | PY | N | PY | N | Y | N | Y | PY | N | N | Y | Y | N | Y | Y |
| Jiang 2015 | Y | 1 | PY | N | PY | N | Y | N | Y | Y | N | Y | Y | Y | Y | Y | Y |
| Kim 2017 | Y | 1 | PY | N | PY | Y | N | PY | Y | Y | N | Y | N | Y | Y | N | Y |
| Kim 2020 | Y | 1 | Y | N | PY | Y | Y | Y | Y | Y | N | Y | N | Y | Y | Y | Y |
| Kishi 2015 | Y | 1 | PY | Y | PY | N | Y | N | Y | Y | Y | Y | N | N | Y | Y | Y |
| Kishi 2017a | Y | 1 | PY | N | Y | Y | Y | N | Y | Y | Y | Y | N | Y | Y | Y | Y |
| Kishi 2017b | Y | 1 | PY | N | Y | Y | Y | N | Y | Y | Y | Y | N | Y | Y | Y | Y |
| Kishi 2018 | Y | 1 | Y | N | Y | Y | Y | PY | Y | Y | Y | Y | N | N | N | Y | Y |
| Kishi 2020 | Y | 1 | PY | N | Y | Y | N | PY | Y | Y | Y | Y | N | Y | Y | Y | Y |
| Kruger 2021 | Y | 1 | Y | N | Y | Y | Y | Y | Y | Y | N | Y | Y | Y | Y | Y | Y |
| Kubo 2015 | Y | 1 | PY | N | PY | N | Y | N | Y | Y | N | Y | Y | Y | Y | Y | Y |
| Lan 2020 | Y | 1 | PY | N | PY | N | Y | N | Y | Y | N | Y | Y | Y | Y | Y | Y |
| Li 2014 | Y | 1 | PY | N | Y | N | Y | N | Y | Y | N | Y | Y | Y | Y | Y | Y |
| Li 2016 | Y | 1 | PY | N | PY | Y | N | N | Y | Y | N | Y | Y | Y | Y | Y | Y |
| Li 2019 | Y | 0 | N | N | PY | N | N | N | Y | N | N | N | N | N | Y | Y | Y |
| Liang 2015 | Y | 1 | PY | N | PY | N | Y | N | Y | Y | N | Y | Y | Y | N | Y | Y |
| Liao 2015 | Y | 1 | N | N | PY | N | Y | N | Y | N | N | Y | N | N | Y | Y | Y |
| Liao 2020 | Y | 1 | PY | N | PY | N | N | Y | Y | Y | N | Y | Y | Y | Y | Y | Y |
| Lin 2019 | Y | 1 | PY | N | PY | N | Y | N | Y | Y | N | Y | N | N | Y | N | Y |
| Liu 2015 | Y | 1 | PY | N | PY | Y | Y | N | Y | Y | N | Y | N | N | N | Y | Y |
| Liu 2019 | Y | 1 | N | N | Y | N | Y | N | Y | N | N | Y | N | N | Y | Y | Y |
| Lu 2020 | Y | 1 | PY | N | PY | Y | Y | N | Y | Y | N | Y | N | Y | Y | Y | Y |
| Ma 2014 | Y | 1 | PY | N | Y | Y | N | Y | Y | Y | N | Y | Y | Y | Y | Y | Y |
| Ma 2018 | Y | 1 | PY | Y | Y | N | Y | N | Y | Y | Y | Y | Y | Y | Y | Y | Y |
| Manolopoulos 2019 | Y | 1 | Y | N | Y | Y | Y | N | Y | N | N | Y | Y | N | Y | Y | Y |
| Matsunaga 2015a | Y | 1 | PY | N | PY | Y | Y | N | Y | Y | N | Y | N | Y | Y | Y | Y |
| Matsunaga 2015b | Y | 1 | PY | N | Y | N | Y | N | Y | Y | N | Y | N | N | Y | Y | Y |
| Matsunaga 2015c | Y | 1 | PY | N | PY | Y | Y | N | Y | Y | Y | Y | N | Y | Y | Y | Y |
| Matsunaga 2016 | Y | 1 | PY | N | Y | Y | Y | N | Y | Y | Y | Y | N | Y | Y | Y | Y |
| Matsunaga 2019a | Y | 1 | PY | N | Y | Y | Y | N | Y | Y | Y | Y | N | Y | Y | Y | Y |
| Matsunaga 2019b | Y | 1 | PY | N | Y | Y | Y | N | Y | Y | Y | Y | Y | Y | Y | Y | Y |
| May 2018 | Y | 1 | Y | N | PY | N | Y | N | Y | Y | N | Y | Y | Y | Y | Y | Y |
| McCleery 2020 | Y | 1 | Y | N | Y | Y | Y | Y | Y | Y | Y | Y | N | Y | Y | Y | Y |
| McGuinness 2014 | Y | 1 | PY | N | Y | Y | N | Y | Y | Y | Y | Y | Y | Y | Y | Y | Y |
| McShane 2019 | Y | 1 | Y | Y | Y | Y | Y | Y | Y | Y | Y | Y | Y | Y | Y | Y | Y |
| Miguel-Alvarez 2015 | Y | 1 | PY | N | PY | Y | N | Y | Y | Y | N | Y | N | N | Y | Y | Y |
| Okuya 2018 | Y | 1 | PY | N | Y | Y | Y | N | Y | Y | Y | Y | N | N | Y | Y | Y |
| Onakpoya 2017 | Y | 1 | PY | N | PY | Y | Y | Y | Y | Y | Y | Y | Y | Y | Y | Y | Y |
| O'Regan 2015 | Y | 1 | PY | N | Y | N | Y | N | Y | Y | N | Y | Y | Y | Y | Y | Y |
| Orgeta 2017 | Y | 1 | PY | N | Y | Y | Y | Y | Y | Y | N | Y | N | Y | Y | Y | Y |
| Penninkilampi 2017 | Y | 1 | PY | N | PY | N | N | N | Y | Y | Y | Y | Y | Y | Y | Y | Y |
| Rao 2014 | Y | 1 | PY | N | PY | Y | N | N | Y | PY | N | Y | N | N | N | N | Y |
| Ruthirakuhan 2018 | Y | 1 | Y | N | Y | Y | Y | Y | Y | Y | Y | Y | Y | Y | Y | Y | Y |
| Ruthirakuhan 2019 | Y | 1 | PY | N | PY | Y | Y | N | Y | Y | N | Y | N | N | Y | Y | Y |
| Sampson 2014 | Y | 1 | Y | N | Y | Y | N | Y | Y | Y | N | Y | N | Y | Y | Y | Y |
| Schmidt 2015 | Y | 1 | PY | N | PY | N | Y | Y | Y | Y | N | Y | Y | Y | N | Y | Y |
| Sepehry 2017 | Y | 1 | N | N | Y | Y | Y | N | Y | N | N | Y | N | N | Y | N | Y |
| Strohle 2015 | Y | 1 | Y | N | PY | Y | Y | N | Y | Y | N | Y | Y | Y | N | Y | Y |
| Su 2015 | Y | 1 | N | N | PY | N | N | N | Y | N | N | Y | N | N | N | Y | Y |
| Tan 2014 | Y | 1 | PY | N | Y | N | Y | N | Y | PY | Y | Y | N | Y | Y | Y | Y |
| Tan 2015a | Y | 1 | PY | N | Y | Y | Y | N | Y | PY | N | Y | N | N | Y | Y | Y |
| Tan 2015b | Y | 1 | PY | N | Y | N | N | N | Y | N | N | Y | N | Y | Y | N | Y |
| Tsoi 2016 | Y | 1 | PY | N | Y | N | Y | N | Y | Y | N | Y | Y | Y | N | N | Y |
| Wang 2015 | Y | 1 | PY | N | PY | Y | Y | N | Y | Y | N | Y | N | N | Y | Y | Y |
| Wang 2016 | Y | 0 | PY | N | PY | Y | Y | N | Y | Y | N | N | N | Y | N | Y | Y |
| Wang 2017 | Y | 1 | PY | N | PY | N | Y | N | Y | Y | N | Y | Y | Y | Y | Y | N |
| Wang 2020a | Y | 1 | PY | N | PY | Y | Y | N | Y | Y | N | Y | N | N | Y | Y | Y |
| Wang 2020b | Y | 1 | PY | N | PY | Y | Y | N | Y | Y | N | Y | N | N | Y | Y | Y |
| Wang 2020c | Y | 1 | PY | N | PY | N | N | N | Y | Y | N | Y | N | N | N | Y | Y |
| Wang 2020d | Y | 1 | PY | N | Y | Y | Y | N | Y | Y | N | Y | N | Y | Y | Y | Y |
| Wei 2020 | Y | 1 | PY | N | Y | N | Y | N | Y | Y | N | Y | N | Y | Y | Y | Y |
| Xing 2014 | Y | 1 | PY | N | PY | Y | Y | Y | Y | Y | N | Y | Y | Y | Y | Y | Y |
| Xu 2015 | Y | 1 | PY | N | Y | N | Y | N | Y | Y | N | Y | N | N | Y | Y | Y |
| Xuan 2020 | Y | 1 | PY | N | PY | Y | Y | N | Y | Y | N | Y | Y | N | Y | Y | Y |
| Yang 2014 | Y | 1 | PY | N | PY | Y | N | N | Y | Y | N | Y | N | Y | Y | Y | Y |
| Yang 2016 | Y | 1 | PY | N | PY | Y | N | N | Y | Y | N | Y | N | Y | Y | Y | Y |
| Zeng 2015 | Y | 1 | PY | N | Y | Y | Y | N | Y | Y | N | Y | N | Y | Y | Y | Y |
| Zhang 2016a | Y | 1 | PY | Y | PY | N | Y | N | Y | Y | N | Y | N | Y | Y | Y | Y |
| Zhang 2016b | Y | 1 | PY | N | PY | N | N | N | Y | N | N | Y | N | N | N | Y | Y |
| Zhang 2019 | Y | 1 | PY | N | Y | Y | Y | N | Y | Y | N | Y | N | Y | N | Y | Y |
| Zhou 2015 | Y | 1 | Y | N | Y | Y | Y | N | Y | Y | N | Y | Y | Y | Y | Y | Y |
| Zhou 2017 | Y | 1 | PY | N | PY | N | Y | N | Y | Y | N | Y | N | Y | Y | Y | N |
| Zhou 2020 | Y | 1 | PY | N | PY | N | N | N | Y | Y | N | Y | Y | Y | Y | Y | Y |
| Zhu 2015 | Y | 1 | PY | N | PY | N | Y | N | Y | Y | N | Y | N | N | N | Y | Y |
| Zhu 2019 | Y | 1 | PY | N | PY | N | Y | Y | Y | Y | N | Y | N | Y | Y | Y | Y |

Key: Y: Yes. PY: Partially yes. N: No. * Critical domains of AMSTAR 2 include item 2, item 4, item 7, item 9, item 11, item 13, item 15. For item 15, if the missing of test for publication bias is because of less than 10 individual studies, we will not consider it as a critical flaw. Detailed information regarding each item of AMSTAR2 and each study could be found in Appendix 3-4.

# Appendix 6a Associations between bibliographical characteristics of systematic reviews and individual AMSTAR-2 item performance - results of binary logistic regression

| AMSTAR2 item (Dependent variable) | Predictor | AOR (95%CI) | *p* ^a^ |
| --- | --- | --- | --- |
| 3. Did the review authors explain their selection of the study designs for inclusion in the review? | Cochrane reviews ^b^ | 13.0(1.84,91.8) | 0.010* |
| 10. Did the review authors report on the sources of funding for the studies included in the review? | Cochrane reviews ^b^ | 30.6(3.58, 261.4) | 0.002** |
| 12. If meta-analysis was performed, did the review authors assess the potential impact of RoB in individual studies on the results of the meta-analysis or other evidence synthesis? | Corresponding author from Asia ^c^  Pharmacological treatments ^d^  Both types ^d^ | 0.19(0.06,0.62)  7.44(1.49,37.3)  16.5(1.44,188.5) | 0.007**  0.015*  0.024* |
| 14. Did the review authors provide a satisfactory explanation for, and discussion of, any heterogeneity observed in the results of the review? | Higher impact factor | 1.50(1.04,2.18) | 0.032* |

Notes: In the model, dependent variable is the rating for each item (Yes/No).

CI, confidence interval; AOR, adjusted odds ratio; ^a^ *P* values for all Hosmer–Lemeshow tests were >0.1, indicating good model fit for all logistic regression analyses. ^b^ Non-Cochrane reviews were used as reference. ^c^ SRs led by corresponding author from Europe were used as reference. ^d^ SRs of non-pharmacological interventions were used as reference.

# Appendix 6b Associations between bibliographical characteristics of systematic reviews and individual AMSTAR-2 item performance - results of multinomial logistic regression

| AMSTAR2 item (Dependent variable) | Predictors¶ | AOR(95%CI)_1/3_ | AOR(95%CI)_2/3_ |
| --- | --- | --- | --- |
| 2. Did the report of the review contain an explicit statement that the review methods were established prior to the conduct of the review and did the report justify any significant deviations from the protocol? | Year of publication: 2019-2021^a^  Corresponding author from Asia ^b^  Treatment types: Both types^c^ | -  -  - | 0.11(0.02,0.77)**  7.13(1.17,43.6)**  0.05(0.004,0.80)** |
| 4. Did the review authors use a comprehensive literature search strategy? | Corresponding author from America ^b^  Pharmacological treatments ^c^ | -  - | 0.04(0.002, 0.63)**  0.11(0.02, 0.69)** |
| 7. Did the review authors provide a list of excluded studies and justify the exclusions? | Corresponding author from Asia ^b^  Corresponding author from America ^b^  Pharmacological treatments ^c^ | 55.7(3.83,809.3)**  55.2(2.37, 1282.4)**  0.03(0.001, 0.77)* | -  -  - |

Notes: In the model, dependent variable is the rating for each item (No = 1; Partial yes = 2; Yes = 3(reference)).

¶: *P* >0.1 for all Pearson test and deviance test, and *p*<0.05 for all likelihood ratio tests, indicating good model fits for all multinomial regression analysis.

CI, confidence interval; AOR, adjusted odds ratio; *: *p*<0.05; **: *p*<0.01.

^a^: SRs published in 2014-2015 were used as reference. ^b^ SRs led by corresponding author from Europe were used as reference. ^c^ SRs of non-pharmacological interventions were used as reference.
